# Patterns, management, and outcomes of traumatic pelvic fracture: insights from a multicenter study

**DOI:** 10.1186/s13018-020-01772-w

**Published:** 2020-07-09

**Authors:** Husham Abdelrahman, Ayman El-Menyar, Holger Keil, Abduljabbar Alhammoud, Syed Imran Ghouri, Elhadi Babikir, Mohammad Asim, Matthias Muenzberg, Hassan Al-Thani

**Affiliations:** 1 Trauma Surgery, Department of Surgery, Hamad General Hospital, Doha, Qatar; 2 Clinical Research, Trauma & Vascular Surgery, Department of Surgery, Hamad General Hospital, Doha, Qatar; 3 Department of Clinical Medicine, Weill Cornell Medical School, Doha, Qatar; 4 Department for Trauma and Orthopaedic Surgery, BG Trauma Center Ludwigshafen at Heidelberg University Hospital, Ludwig-Guttmann-Strasse 13, 67071 Ludwigshafen am Rhein, Germany; 5 Orthopedic Surgery, Department of Surgery, Hamad General Hospital, Doha, Qatar; 6 Trauma & Vascular Surgery, Department of Surgery, Hamad General Hospital, Doha, Qatar

**Keywords:** Pelvic fracture, Hemodynamic instability, Tiles’s classification, Mortality

## Abstract

**Background:**

Traumatic pelvic fracture (TPF) is a significant injury that results from high energy impact and has a high morbidity and mortality.

**Purpose:**

We aimed to describe the epidemiology, incidence, patterns, management, and outcomes of TPF in multinational level 1 trauma centers**.**

**Methods:**

We conducted a retrospective analysis of all patients with TPF between 2010 and 2016 at two trauma centers in Qatar and Germany.

**Results:**

A total of 2112 patients presented with traumatic pelvic injuries, of which 1814 (85.9%) sustained TPF, males dominated (76.5%) with a mean age of 41 ± 21 years. In unstable pelvic fracture, the frequent mechanism of injury was motor vehicle crash (41%) followed by falls (35%) and pedestrian hit by vehicle (24%). Apart from both extremities, the chest (37.3%) was the most commonly associated injured region. The mean injury severity score (ISS) of 16.5 ± 13.3. Hemodynamic instability was observed in 44%. Blood transfusion was needed in one third while massive transfusion and intensive care admission were required in a tenth and a quarter of cases, respectively. Tile classification was possible in 1228 patients (type A in 60%, B in 30%, and C in 10%). Patients with type C fractures had higher rates of associated injuries, higher ISS, greater pelvis abbreviated injury score (AIS), massive transfusion protocol activation, prolonged hospital stay, complications, and mortality (*p* value < 0.001). Two-thirds of patients were managed conservatively while a third needed surgical fixation. The median length of hospital and intensive care stays were 15 and 5 days, respectively. The overall mortality rate was 4.7% (86 patients).

**Conclusion:**

TPF is a common injury among polytrauma patients. It needs a careful, systematic management approach to address the associated complexities and the polytrauma nature.

## Introduction

In polytrauma patients, pelvic injuries are commonly seen. Pelvic injuries range from minor lacerations to major fractures that may be devastating and complex. Injury to pelvic region accounts for 10% of all the blunt trauma admissions [1]. Population-based studies reported the average prevalence of pelvic fractures to be 20 per 100,000 individuals [2, 3]. The clinical presentation and outcomes of pelvic fractures depend on the hemodynamic status. Managing these injuries is challenging both from the diagnostic and therapeutic perspectives, especially in unstable patients. Despite the trend toward initial selective imaging, current recommendation favors routine pelvic X-ray in blunt trauma as an initial screening tool to rule out pelvic fractures [4]. Blunt traumatic injuries secondary to motor vehicle crashes (cars and motorcycles), pedestrian and bicycle-hits by vehicles, and falls from height are the main mechanisms of pelvic injuries. Usually, young men are more susceptible to the high-energy traumatic injuries [3]. Such high-energy mechanisms are most commonly associated with pelvic fractures, but still, low-energy trauma may lead to a fracture in some patients, particularly among the elderly [5, 6].

Moreover, high-impact pelvic fractures may also present with other associated injuries, particularly peripelvic soft tissue injuries, extremity fractures, abdominal solid organ injuries (SOIs), and injuries to the chest [1]. The severity of pelvic injury also dictates the overall injury severity, which might result in higher mortality [7, 8]. The reported rate of in-hospital mortality in pelvic fracture usually ranges from 5–20% but may go up to 50% in cases with open compound fractures. This high mortality is mainly attributed to the hemodynamic instability resulted from exsanguinating hemorrhage in young patients and multiorgan failure in elderly patients [9]. The various sources of hemodynamic instability include the disruption of venous and arterial vessels near the fracture, the exposed fracture ends, the associated soft-tissue injury, and SOIs [3, 7, 10]. The admission of unstable patients with pelvic fractures represents a complex life-threatening scenario which necessitates early aggressive resuscitation and prompt surgical intervention [11]. It is pertinent to deal with the pelvis as a “visceral organ” with multiple sources of bleeding [12]. Reports of pelvic fracture are limited and diverse in both the features and outcomes. A better characterization of pelvic fracture is important to guide the decision makers. The present multicenter study aims to describe the common patterns of pelvic fractures, hemodynamic status, management, and clinical outcomes in patients with a traumatic pelvic fracture from two level 1 trauma centers of different continents.

## Methods

### Study design

A retrospective cohort study was conducted for all patients who sustained traumatic pelvic injuries and were admitted at two trauma centers: Hamad Trauma Center (HTC), the level 1 national trauma center in the state of Qatar, and BG Trauma Center Ludwigshafen, Germany, (level 1 trauma center) between January 2010 and June 2016. The final analysis comprised of all patients with traumatic pelvic fractures (*n* = 1814). We have excluded patients presented with cardiac arrest on arrival at the hospital and those without pelvic fracture. The study was approved by the institutional review board (IRB) of the Medical Research Center at Hamad Medical Corporation [HMC IRB# 14175/14 & 16395/16; BG IRB# 837.500.17 (11334)] with a waiver of informed consent. In Qatar, data were retrospectively obtained from a prospectively maintained trauma registry database of the HTC. HTC is a level 1 accredited center by the Accreditation Canada; it is a tertiary hospital with a dedicated trauma team of surgeons and intensivist, immediate access to care, operating theater, interventional radiology, massive transfusion protocol, and advanced prehospital care. HTC data repository with uniform data elements are reporting to the National Trauma Data Bank (NTDB) and the Trauma Quality Improvement Program (TQIP) of the American College of Surgeons-Committee on Trauma (ACS-COT). The HTC is the only tertiary care facility in the country to which around 1500–1700 trauma patients are admitted annually. Therefore, the data obtained from the Qatar trauma registry are nationally representative which covers a population of approximately 2.6 million. The German center participates in the German Trauma [2018: 193 trauma room admissions, 125 patients injury severity score ((ISS) > 16)] as well as the pelvic injury registry). The German center serves a population of about 1.5 million people in the metropolitan area Rhein-Neckar. The German data were retrospectively obtained from the clinic information system as well as from a prospectively maintained trauma database. The BG Trauma Center Ludwigshafen is a professional accident clinic in Ludwigshafen. The primary focus of this center includes trauma surgery and orthopedics, plastic and reconstructive surgery, and hand and tumor surgery. From 1997, the BG hospital has initiated academic research and teaching, and the management of patients with trauma, hand, plastic, and burn surgery.

The trauma teams assessed all the pelvic trauma patients, and pelvic binder is used whenever indicated. Initial assessment and management are following the ATLS guidelines. All patients get a pelvic X-ray and a CT scan if hemodynamically stable. Patient management is carried out by a multidisciplinary team lead by trauma surgeons and included intensivist, anesthesiologist, orthopedic surgeons, other surgical subspecialties according to the associated injuries, and radiologist.

### Data collection

**Fig. 1:**
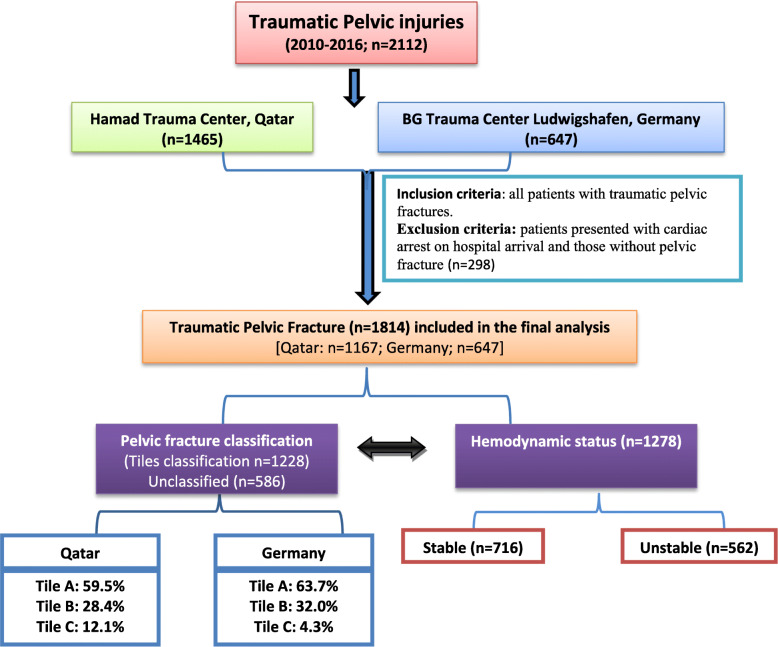
Flow diagram showing study design

Data were retrieved for demographic characteristics (age and gender); mechanisms of injuries; associated injuries including injuries to the head, chest, abdomen, spine, upper and lower extremity; injury characteristics such as Glasgow Coma Score at emergency department (ED); Abbreviated Injury Score (AIS); Injury Severity Score (ISS); and Revised Trauma Score (RTS), initial vitals at ED such as respiratory rate, oxygen saturation (SpO2), systolic blood pressure (SBP), diastolic blood pressure (DBP), heart rate, and shock Index (SI); ED disposition; pelvic fracture pattern (Modified Tile’s AO Müller classification); need for blood transfusion; number of blood units transfused; massive transfusion protocol (MTP) activation; surgical intervention (open reduction and internal fixation, closed reduction and external fixation); in-hospital complications [pneumonia, sepsis, multiorgan dysfunction (acute respiratory distress syndrome (ARDS), and acute kidney injury (AKI)), deep venous thrombosis (DVT), and pulmonary embolism (PE)]; ventilator days; length of intensive care unit; and hospital stays as well as in-hospital mortality. We excluded patients who were brought in dead and those who had non-fracture soft tissue pelvic injury or a dislocated hip (Fig. 1).

The fracture patterns for the pelvis and acetabular fractures were classified according to the modified Tile AO Müller classification by experienced orthopedic surgeons [13, 14], which categorizes pelvic fractures into three main types based on stability and integrity of the posterior sacroiliac complex. It also takes into consideration the direction of the traumatic force resulting in pelvic fracture. In type A fracture (stable), the fracture does not involve the posterior arch. Type B fracture is a result of rotational forces that cause partial disruption of the posterior sacroiliac complex, considered partially stable (rotationally unstable). Complete disruption of the posterior complex (including the sacrospinous and sacrotuberous ligaments) occurs in type C fractures that are both rotationally and vertically unstable. Posterior injuries more commonly result from high impact and cause a lot of tissue disruptions and potential significant bleeding. Cases with difficult or overlapping classes were considered unclassified (almost one-third of the cohort) and were not included in the final analysis.

Shock index (SI) was calculated as HR/SBP recorded at the time of presentation to the emergency department [15]. Either of three parameters determined the hemodynamic stability (SBP ≤ 90 mmHg and/or HR ≥ 120 and/or SI ≥ 0.8).

### Statistical analysis

Data were presented as proportions, medians, range, and means ± standard deviation, as appropriate. Data were compared for the pattern of pelvic fracture (Tile A vs. Tile B vs. Tile C) and hemodynamic status (stable versus unstable). Differences in categorical variables between respective groups were analyzed using the chi-square test. The continuous variables were analyzed using Student’s *t* test and one-way ANOVA, as appropriate. Yates’ corrected chi-square was used for categorical variables if the expected cell frequencies were below 5; for continuous skewed data non-parametric, Mann-Whitney test was performed. A two-tailed *P* value of < 0.05 was considered to be statistically significant. Data analysis was carried out using the Statistical Package for the Social Sciences, version 18 (SPSS, Inc, Chicago, IL).

## Results

This was a retrospective observational study for cases of pelvic fracture admitted to two participating centers between January 2010 and June 2016. Figure 1 shows the overall study design. We identified 2112 patients who sustained traumatic pelvic injuries, of which 1814 (86%) had traumatic pelvic fractures. This number represents 11% of the total trauma admissions in Qatar and 13% of the total trauma admissions in the German center during the study period. Table 1 shows the demographic characteristics, mechanisms of injury, associated injuries, injury severity scores, vital signs including oxygen saturation on admission, ED dispositions, and outcomes of pelvic fracture. The mean age for the whole sample was 41 ± 21 years (32 ± 14 in Qatar and 57.4 ± 21.6 in the German center).The gender showed that the majority (76.5%) were males (88% in Qatar and 55% in the German center); the male to female ratio was 3:1. The most common mechanisms of pelvic fracture were traffic-related in 59% of all cases, followed by fall from height (33%). In unstable cases, the frequent mechanism of injury was MVC (41%) followed by falls (35%) and pedestrian hit by vehicle (24%).

**Table 1:** Overall demographic characteristics, clinical presentation, and outcome of patients with pelvic fracture (*n* = 1814)

Variables	Value
**Age (mean ± SD) years**	41.2 ± 21.1
**Males**	1387 (76.5%)
**Females**	426 (23.5%)
**Mechanism of injury**	
Fall from height	595 (32.8%)
Motor vehicle crashes	506 (27.9%)
Pedestrian hit	508 (28.0%)
All-terrain vehicle crashes	28 (1.5%)
Motorcycle crashes	16 (0.9%)
Bicycle crashes	11 (0.6%)
Hit by a falling object	119 (6.6%)
Self-inflicted injuries	10 (0.6%)
Others	21 (1.2%)
**Associated injuries**	
Chest	677 (37.3%)
Spine	573 (31.6%)
Abdomen	497 (27.4%)
Head	330 (18.2%)
Lower extremity	462 (25.5%)
Upper extremity	487 (26.8%)
**Glasgow coma score at admission (mean ± SD) (*n*= 1466)**	13.3 ± 3.9
**Injury severity score (mean ± SD)**	16.5 ± 13.3
**Revised trauma score (mean ± SD) (*n*= 1200)**	7.23 ± 1.38
**Pelvis AIS (mean ± SD)**	2.4 ± 0.7
**Head AIS (mean ± SD)**	3.3 ± 1.3
**Chest AIS (mean ± SD)**	2.8 ± 0.8
**Abdomen AIS (mean ± SD)**	2.7 ± 1.1
**SBP at ED (*n*= 1295) (mean ± SD)**	120.2 ± 21.7
**DBP at ED (*n*= 1221) (mean ± SD)**	73.1 ± 15.8
**Pulse rate at ED** (mean ± SD) (*n* = 1306)	95.8 ± 23.1
**Oxygen saturation at ED** (mean ± SD) (*n* = 1282)	97.7 ± 6.3
**Respiratory rate at ED** (mean ± SD) (*n* = 1208)	19.5 ± 4.5
**Hemodynamic instability**	562 (44.0%)
**ED disposition** (*n* = 1806)	
Admission to intensive care unit	398 (22.0%)
Operating room	342 (18.9%)
In-hospital wards	1061 (58.7%)
High dependency unit	5 (0.3%)
**Number of blood units transfused (median, range)**	6 (1–122)
**Patients required blood transfusion**	625 (34.5%)
**Massive transfusion (*n*= 885)**	94 (10.6%)
**Endotracheal intubation (*n*= 885)**	203 (22.9%)
**FAST positive (*n*= 833)**	92 (11.0%)
**Management (*n*= 1829)**	
Conservative management	1172 (64.6%)
Surgical intervention*	642 (35.4%)
**In-hospital complications**	
Pneumonia	118 (6.5%)
Sepsis	57 (3.2%)
Acute respiratory distress syndrome (ARDS)	54 (3.0%)
Acute kidney injury (AKI)	44 (2.4%)
Deep vein thrombosis (*n* = 885)	7 (0.8%)
Pulmonary embolism (*n* = 885)	4 (0.5%)
Multiorgan failure (*n* = 885)	6 (0.7%)
**Needed mechanical ventilation (median, range) days**	5 (1–63)
**Intensive care unit length of stay (median, range)**	5 (1–74)
**Hospital length of stay (median, range)**	15 (1–505)
**Mortality**	86 (4.7%)

*ED* emergency department, *SBP* systolic blood pressure, *DBP* diastolic blood pressure, *AIS* abbreviated injury score

*Open reduction and internal fixation, closed reduction and external fixation

**Fig. 2:**
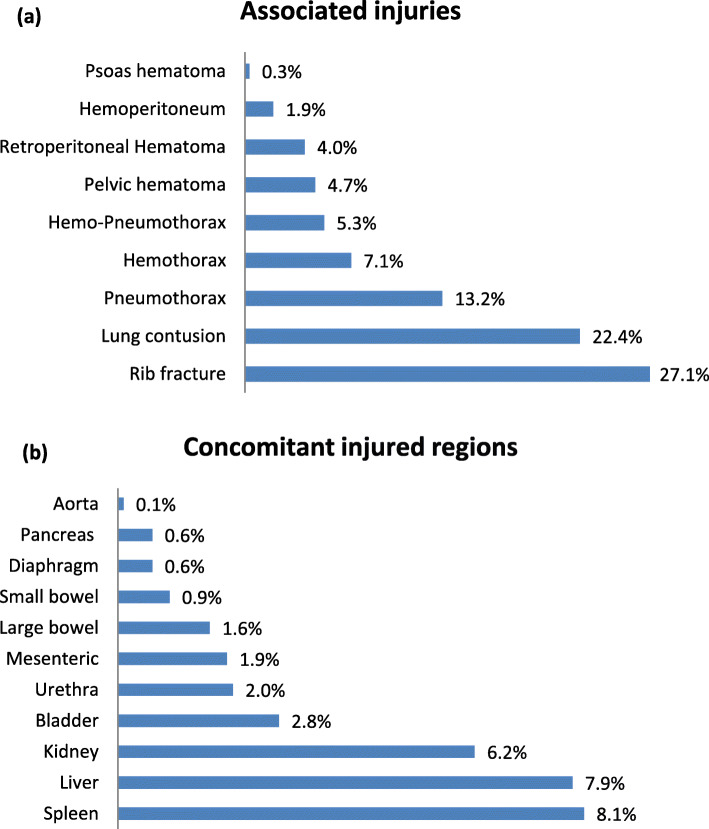
Distribution of **a** associated injuries and **b** concomitant injured regions with pelvic fracture (*n* = 885)

The associated injury by region involved the chest (37%) followed by the spine (32%), abdomen (27%), upper extremities (27%), and lower extremities (26%) while the head injuries were associated in 18% of cases. However, if lower and upper extremity percentages are combined, they would represent 53% of the cohort and thus represent the most common association. Figure 2 demonstrates the distribution of associated specific injuries with pelvic fracture.

The mean ISS was 16.5 ± 13.3 (15.8 ± 10.6 in Qatar and 17.7 ± 16.9 in the German center); RTS was 7.23 ± 1.38; the head AIS was 3.3 ± 1.3. The majority were presented with blood pressure and oxygen saturation within the normal range but with a high mean heart rate (96 beat/min) and respiratory rate of 20 breaths per minute. Positive FAST was seen in 11%. Blood transfusion was needed in 34.5% (38% in Qatar and 28% in the German center), and in 11%, it reached the massive transfusion limit of 10 units over 24 h. The majority of fractures (65%) were treated conservatively, while 35% underwent surgical treatment (reduction and fixation open or closed external fixation).

Concerning ED disposition, 59% of patients were admitted to regular trauma ward and a quarter needed trauma ICU admission, and 19% needed immediate transfer to the operating room for life-saving interventions.

The observed in-hospital complications included pneumonia (6.5%), sepsis (3%), ARDS (3%), and AKI (2.4%). The frequency of other complications such as DVT (1%), PE (0.5%), and multi-organ failure (1%) was very low. The median length of mechanical ventilation and ICU stay was 5 days, and the median hospital stay was 15 days. Eighty-six patients died with an overall in-hospital mortality of 4.7% (5% in Qatar and 4% in Germany).

Table 2 shows the clinical characteristics and outcomes by types of pelvic fractures. The pelvic fracture pattern based on Tiles classification was available in 1228 (68%). Tile A (60%) was most frequently observed, followed by Tile B (30%) and Tile C (10%). Two hundred eighty-four patients were having acetabular fractures, of which 273 were isolated acetabular fractures, and hip dislocation was observed in 8 patients.

**Table 2:** Clinical characteristics and outcome by types of pelvic fractures (Tile classification; *n* = 1228)

	Tile A (***n*** = 745; 60.7%)	Tile B (***n*** = 361; 29.4%)	Tile C (***n*** = 122; 9.9%)	***P*** value
**Age (mean ± SD) years**	41.5 ± 21.7	38.8 ± 19.6	36.1 ± 14.9	0.009
**Males**	553 (74.3%)	270 (74.8%)	100 (82.0%)	0.18
**Mechanism of injury**				
Fall from height	250 (33.6%)	118 (32.7%)	40 (32.8%)	0.001 for all
Motor vehicle crashes	192 (25.8%)	104 (28.8%)	31 (25.4%)	
Pedestrian hit	248 (33.3%)	92 (25.5%)	27 (22.1%)	
Hit by a falling object	32 (4.3%)	39 (10.8%)	21 (17.2%)	
Others	23 (3.1%)	8 (2.2%)	3 (2.5%)	
**Associated injuries**				
Chest	249 (33.4%)	130 (36.0%)	55 (45.1%)	0.04
Spine	236 (31.7%)	115 (31.9%)	64 (52.5%)	0.001
Abdomen	182 (24.4%)	134 (37.1%)	68 (55.7%)	0.001
Head	131 (17.6%)	70 (19.4%)	26 (21.3%)	0.53
Lower extremity	207 (27.8%)	91 (25.2%)	36 (29.5%)	0.55
Upper extremity	170 (22.8%)	93 (25.8%)	39 (32.0%)	0.07
**Injury severity score (mean ± SD)**	13.7 ± 10.7	16.9 ± 11.6	23.7 ± 15.3	0.001
**Glasgow coma score at admission (mean ± SD) (*n*= 1017)**	13.7 ± 3.5	13.0 ± 4.2	12.8 ± 4.2	0.01
**Revised trauma score (mean ± SD) (*n*= 874)**	7.4 ± 1.2	7.2 ± 1.4	6.9 ± 1.7	0.004
**Pelvis AIS (mean ± SD)**	2.1 ± 0.4	2.5 ± 0.7	2.9 ± 1.0	0.001
**Chest AIS (mean ± SD) (*n*= 434)**	2.7 ± 0.8	2.8 ± 0.8	3.0 ± 0.9	0.04
**Abdomen AIS (mean ± SD) (*n*= 384)**	2.5 ± 1.1	2.4 ± 0.8	2.7 ± 1.0	0.30
**Patients required blood transfusion**	187 (25.1%)	146 (40.4%)	88 (72.1%)	0.001
**Blood units transfused (median, range)**	4 (1–83)	6 (1–48)	8 (1–55)	0.008
**MTP (blood units > 10) (*n*= 629)**	33 (7.5%)	19 (16.4%)	26 (35.6%)	0.001
**Intubation (*n*= 629)**	85 (19.3%)	37 (31.9%)	32 (43.8%)	0.001
**FAST positive (*n*= 591)**	44 (10.8%)	19 (16.7%)	13 (19.1%)	0.06
**Shock Index (*n*= 913)**				
< 0.8	326 (61.5%)	145 (52.9%)	42 (38.5%)	0.001 for all
≥ 0.8	204 (38.5%)	129 (47.1%)	67 (61.5%)	
**Management**				
Conservative	573 (76.9%)	259 (71.7%)	65 (53.3%)	0.001 for all
Surgical intervention	172 (23.1%)	102 (28.3%)	57 (46.7%)	
**In-hospital complications**				
Pneumonia	35 (4.7%)	34 (9.4%)	9 (7.4%)	0.01
Sepsis	13 (1.7%)	18 (5.0%)	7 (5.7%)	0.003
ARDS	8 (1.1%)	8 (2.2%)	9 (7.4%)	0.001
Acute kidney injury	9 (1.2%)	6 (1.7%)	11 (9.0%)	0.001
Deep vein thrombosis (*n* = 629)	3 (0.7%)	0 (0.0%)	3 (4.1%)	0.01
Pulmonary embolism (*n* = 629)	2 (0.5%)	0 (0.0%)	1 (1.4%)	0.40
Organ failure (*n* = 629)	1 (0.2%)	1 (0.9%)	2 (2.7%)	0.04
**Ventilatory days (median, range)**	6 (1–53)	5.5 (1–63)	8 (1–49)	0.91
**ICU length of stay (median, range)**	4 (1–71)	5 (1–74)	5 (1–61)	0.62
**Hospital length of stay (median, range)**	11 (1–505)	18 (1–257)	28 (1–165)	0.001
**Mortality**	24 (3.2%)	17 (4.7%)	16 (13.1%)	0.001

*AIS* abbreviated injury score, *MTP* massive transfusion protocol

Falls were the most common mechanism of injury in all pelvic ring fracture types, followed by pedestrians hit by cars. MVC and falls were the most common involved mechanisms in type A pelvic fractures. The MVC was the most commonly observed mechanism in patients with type B (*P* value = 0.001).

The associated injuries including chest, spine, and abdomen showed a significant association with type C fractures (*P* value = 0.001). Also, patients with Tile C were more likely to have higher ISS, pelvis AIS, chest AIS, abdomen AIS, and lower admission GCS in comparison to Tile A and B (*P* value = 0.001).

Elevated shock index (≥ 0.8) was found in 61.5% of Tile C compared to 47% in Tile B and 38.5% in Tile A (*P* value = 0.001). The need for blood transfusion (*P* value = 0.001), MTP (*P* value = 0.001), intubation (*P* value = 0.001), and surgical intervention (*P* value = 0.001) were also greater in patients with Tile C.

Concerning in-hospital complications, patients with Tile B and C showed a higher association with pneumonia (*P* value = 0.01), whereas the rate of sepsis, ARDS, AKI, and DVT were significantly higher in Tile C group (*P* value = 0.001 for all). Also, patients with Tile C had prolonged hospital stay (*P* value = 0.001) with higher in-hospital mortality (13%) compared to Tile A (3%) and Tile B (5%); *P* value = 0.001.

Table 3 compares the clinical characteristics and outcomes of pelvic fracture by hemodynamic status (stable vs unstable). The majority of males fell in the stable group while the majority of females were in the unstable group. Hemodynamically unstable patients tended to be younger, sustained more associated injuries, severely injured (higher ISS, higher AIS, lower GCS), and lower RTS as compared to stable patients (*P* value = 0.001). Also, hemodynamically unstable patients were more likely to have unstable pelvic fractures, i.e., Tile B and C, and had higher rates of intubation, positive FAST, in-hospital complications, blood transfusion, and MTP and had prolonged mechanical ventilation, ICU and hospital stay than the stable group (*P* value = 0.001). On the other hand, stable patients were more likely to be male, frequently had Tile A (*P* value = 0.001), and acetabular fracture (*P* value = 0.004) as compared to the unstable group. The rate of mortality was significantly higher in the hemodynamically unstable group (9% vs. 1.4%; *P* value = 0.001).

**Table 3:** Comparison of clinical characteristics and outcome by hemodynamic status of pelvic fracture patients (*n* = 1278)

	Stable (***n*** = 716; 56.0%)	Unstable (***n*** = 562; 44.0%)	***P*** value
**Age (mean ± SD) years**	37.1 ± 15.5	30.9 ± 16.3	0.001
**Males**	637 (89.0%)	455 (81.0%)	0.001
**Associated injuries**			
Chest	247 (34.5%)	300 (53.4%)	0.001
Spine	237 (33.1%)	240 (42.7%)	0.001
Abdomen	194 (27.1%)	251 (44.7%)	0.001
Head	106 (14.8%)	151 (26.9%)	0.001
**Injury severity score (mean ± SD)**	13.9 ± 10.1	21.8 ± 14.2	0.001
**Glasgow coma score at admission (mean ± SD)**	14.1 ± 2.9	12.2 ± 4.8	0.001
**Revised trauma score (mean ± SD)**	7.6 ± 0.9	6.9 ± 1.6	0.001
**Pelvis AIS (mean ± SD)**	2.3 ± 0.5	2.5 ± 0.8	0.001
**Chest AIS (mean ± SD)**	2.7 ± 0.8	2.8 ± 0.8	0.02
**Abdomen AIS (mean ± SD)**	2.4 ± 1.0	2.7 ± 1.0	0.01
**TILE AO Müller classification (*n*= 913)**			
Tile A	322 (64.0%)	208 (50.7%)	0.001 for all
Tile B	141 (28.0%)	133 (32.4%)	
Tile C	40 (8.0%)	69 (16.8%)	
**Acetabulum fracture**	160 (22.3%)	89 (15.8%)	0.004
**Blood transfusion**	176 (24.6%)	354 (63.0%)	0.001
**Blood units (median, range)**	3 (1-38)	6 (1-83)	0.001
**MTP blood units > 10 (*n*= 861)**	4 (0.9%)	77 (19.6%)	0.001
**Intubation (*n*= 861)**	41 (8.7%)	147 (37.5%)	0.001
**FAST positive (*n*= 812)**	26 (5.9%)	62 (16.8%)	0.001
**Management (*n*= 1293)**			
Conservative	449 (62.7%)	342 (60.9%)	0.49 for all
Surgical intervention	267 (37.3%)	220 (39.1%)	
**In-hospital complications**			
Pneumonia	25 (3.5%)	80 (14.2%)	0.001
Sepsis	9 (1.3%)	37 (6.6%)	0.001
ARDS	10 (1.4%)	28 (5.0%)	0.001
Acute kidney injury	6 (0.8%)	26 (4.6%)	0.001
Deep vein thrombosis (*n* = 861)	2 (0.4%)	4 (1.0%)	0.29
Pulmonary embolism (*n* = 861)	2 (0.4%)	2 (0.5%)	0.85
Organ failure (*n* = 861)	1 (0.2%)	4 (1.0%)	0.12
**Ventilatory days (median, range)**	4 (1-49)	7 (1-63)	0.008
**ICU length of stay (median, range)**	4 (1-71)	6 (1-74)	0.001
**Hospital length of stay (median, range)**	13 (1-125)	21 (1-505)	0.001
**Mortality**	10 (1.4%)	51 (9.1%)	0.001

In Qatar, during the study period, angiography and subsequent angioembolization were performed in 65 patients. The most commonly involved vessel was the internal iliac artery (50 cases), while the other embolized vessels were the pudendal, sacral, and other unnamed arteries with immediate satisfactory results and a smooth hospital course. Data on arterial embolization were not available at the German institution (If needed, patient could be transferred to a cooperating hospital and sent back after angioembolization).

## Discussion

The current study is a large multicenter retrospective observational study that describes the epidemiology, clinical presentation, complications, and mortality in patients with pelvic fractures in two trauma centers. The first trauma center in the state of Qatar contributes to 64% of the data while the second center in Germany contributes to 36% of the data.

Pelvic fracture is not uncommon and is nearly reported in 10% (11% in Qatar and 13% in German) of admitted patients and tends to affect young subjects (mean 41 years old) in our cohort. Pelvic fracture caused by traffic-related injuries and falls suggested a high energy impact. In unstable cases, the frequent mechanism of injury was MVC followed by falls and pedestrian hit by vehicle.

Polytrauma is the norm in the present study, with an average ISS of 16. After excluding the extremity injuries, chest injuries outnumbered all other anatomical injuries in nearly 40% of cases in our cohort.

A high index of suspicion and prompt recognition of instability, both hemodynamic and fracture-related mechanical patterns, is of paramount importance in pelvic injuries.

The frequency of pelvic fracture in our cohort is relatively high, which represents the severe nature of trauma in young population, as it has reported in other countries like the UK, Sweden, and Germany [16–18]. However, when comparing nationally based databases, there is variability in the prevalence and the affected age and gender worldwide [2, 3, 6, 7, 16–18]. The German center data showed an older age and slight male predominance over females in comparison to the Qatar cases reflecting the country-based difference in the affected population [18].

Prior data advocated the crucial impact of age on the outcomes in trauma patients as advanced age alters the physiologic status resulting in a suboptimal recovery with higher chances for death and complications [19–23]. However, in the current study, the mean age of patients was 41 years, a unique finding. It represents the national census of Qatar as the majority of population are young expatriate males [24]. This may also explain the possible work-related injury pattern noticed in this cohort as well as the relatively better clinical outcomes in terms of in-hospital complication rates and mortality. The majority of cases had high-energy impacts due to traffic-related injury or falls. Studies have shown that high-energy impacts, particularly road traffic collisions and a pedestrian hit by vehicle, are the primary mechanisms of injuries leading to pelvic fracture [15, 16, 25, 26]. Males are more likely to experience pelvic fractures, as they are more susceptible to these high-energy mechanisms [3]. Furthermore, falls are overrepresented as a leading cause of injury; this finding is exciting and can be explained by work-related falls as Qatar is undergoing a country-wide reconstruction surge in preparation for the World Cup 2022.

In pelvic trauma, the hemodynamic instability on-admission predicts the requirement of massive blood transfusion, injury severity, associated injuries, fracture stability, in-hospital complications, and mortality [21, 25, 27–29]. However, a higher proportion of our patients was hemodynamically stable, admitted to regular trauma wards, and managed conservatively with lower rates of complications and mortality similar to data from the USA and Europe [6, 16–18].

The reported mortality rates in pelvic fracture vary quite widely, which could be as high as 30% [12, 16, 21, 30] or even higher in cases with extensive soft tissue damage [9]. In this cohort, the overall mortality was relatively low 4.7% that was correlated well with reported cases from previous studies in Germany (4%) and the USA (3.5%) [18, 31].

This low mortality may reflect the maturation of the trauma system and improved post-traumatic care with the availability of specialized and multidisciplinary teams, massive transfusion protocol activation, immediate access to the operative room, and interventional radiology as well as subsequent advanced critical care. The basis in many of the contemporary published guideline work group recommendations and performance improvement programs aim to improve the pelvic fracture outcomes [21, 32–34].

The utility of shock index for early predicting significant hemorrhage and timely activation of the trauma team and massive transfusion protocol expedites appropriate care to stop the bleeding and thereby improve clinical outcomes [7, 35–37].

The present study showed higher mortality in unstable pelvic fracture patterns (i.e., Tile C; 13%) as compared to Tile A (3.2%) and B (4.7%). In hemodynamically stable patients, the mortality was 1.4% compared to 9.1% in unstable patients which is similar to the reported rate by Black et al. [36]. The higher mortality in types B and C is contributed to the disruption of the posterior elements and higher rate of bleeding from the rich venous and vascular structures; in type B, the disruption is partial which explains the smaller surge while in type C it is complete disruption [37, 38]. Furthermore, previous German registry data showed a high incidence of complications in the form of sepsis in 5% and multiorgan dysfunction in 25% with a prolonged ICU length of stay [39].

Tile A classification of pelvic fracture is the most common type of pelvic fracture in the present cohort, which is similar to some of the studies that reported stable fractures as the most frequent fracture type [40]. On the other hand, an earlier study from the Netherlands reported Tile B fractures to be predominantly followed by Tile C and Tile A fractures [41].

Agri et al. [42] reported that Tile C fractures were significantly associated with more blood transfusion and a higher rate of mortality as compared to Tile A or B fractures. Unstable pelvic fractures are the most severe skeletal injury due to its complexity, high-energy impact, and potential life threatening bleeding [43–46]. Accurate and prompt assessment of patient injury, physiologic and anatomic classification, and multidisciplinary management approach are essential components for effective management, improved outcomes, and future studies and audits.

### Limitations

We acknowledge the limitations of our study. The retrospective study design and possible bias due to missing information or coding errors are among the most important limitations. Furthermore, the problem of comparability of different centers is another challenge that needs to be addressed. Moreover, trauma patients who died before hospital arrival were not included as well as those who were not admitted and discharged home. The higher frequency of significant associated injuries makes it impossible to separate the mortality caused by pelvic fracture per se effectively. The MTP activation documentation was not complete, so we used blood unit ≥ 10 for the identification of cases that had a massive transfusion. The place of injury was not documented in both centers; therefore, we could not address the work-related injuries. We lack information for arterial embolization from the German institution. Also, our database is lacking results of functional outcomes and chronic sequel such as pain, impotence, and disabilities. These missing pieces of information may urge the need to conduct further studies using isolated pelvic fractures only to determine pelvic fracture-related mortality and other underreported complications of this significant injury to strengthen our findings and to set the optimal time and type of management approaches for the new cases.

However, this is one of the largest databases available, the sample is somewhat homogenous with no wide age variation, and the Qatari center is the national center for trauma care in Qatar, so it is a national representing data. In contrast, the German center covers only 1.5 million populations within Germany.

## Conclusions

This study reveals that pelvic fracture is a common injury among polytrauma patients. Its occurrence and severity vary according to the mechanism of injury. It needs a careful, systematic multispecialty approach to address the associated complexities, mortality, and polytrauma nature.

## Data Availability

Not applicable

## Abbreviations

ISS: Injury severity score
AIS: Abbreviated injury score
MTP: Massive transfusion protocol
SI: Shock index
ED: Emergency department
GCS: Glasgow coma scale
